# Dataset on sustainable construction practices of foreign and indigenous construction firms

**DOI:** 10.1016/j.dib.2018.08.044

**Published:** 2018-08-22

**Authors:** Akinbo Tomisin Faith, Olabosipo I. Fagbenle, Lekan M. Amusan, Afolabi Adedeji

**Affiliations:** Department of Building Technology, Covenant University, College of Science and Technology, Canaanland, Ota, Ogun State, Nigeria

## Abstract

This dataset conducted a comparative analysis of sustainable construction practices of foreign and indigenous construction firms in Lagos state, Nigeria using a structured questionnaire survey. The dataset contains the level of awareness and consistency of practice of sustainable development on construction projects between the foreign and indigenous construction firms and the impact of implementing sustainable development practices. Descriptive analysis such as frequencies, percentage and mean score were used to present the quantitative data in form of tables. Further analysis of the dataset highlight the practices of indigenous and foreign construction firms in sustainable development which can be beneficial to stakeholders in environmental protection and mitigating climate change issues.

**Specifications Table**

**Table:** 

**Subject area**	*Construction*
**More specific subject area**	*Sustainability*
**Type of data**	*Table*
**How data was acquired**	*Survey research design, questionnaire instrument*
**Data format**	*Raw, analyzed*
**Experimental factors**	*Survey of management team of indigenous and foreign construction firms*
**Experimental features**	*Sample selection, frequencies, percentages, mean score and ranking index*
**Data source location**	*Lagos State, Nigeria*
**Data accessibility**	*All the data are in this data article*

**Value of the data**

• The dataset highlights sustainable strategies that can be implemented for creating safe construction activities which are environmentally friendly by construction firms and stakeholders [1], [2], [3], [4], [5]. The scientific community and researchers can use the dataset to measure the compliance and commitment of construction firms in reducing greenhouse gases (GHG).
• The dataset is useful for policy makers in enforcing sustainable practices by construction firms.
• The dataset can be replicated in other sectors to understand the sustainable practices used and the level of awareness in those practices.
• With rising population and the needs to provide adequate housing, high waste generation by the construction needs to be curtailed. Construction clients through this dataset can set limit on waste generation through efficient sustainable development practices.
• Further analysis of the dataset can reveal the rationale of investing in sustainable development practices by indigenous and foreign construction firms.

## 1 Data

The dataset presented in this context described a comparative analysis of sustainable construction practices of foreign and indigenous construction firms in Lagos state, Nigeria. The dataset gives a summary of the construction firms’ information, level of awareness, consistency of practice and the impact of implementing sustainable development practices. The dataset was obtained on a firm-level basis using a primary instrument. Table 1 showed the distribution of the construction firms that participated and adequately filled the structured questionnaire. Table 1 showed that 27 (67.5%) of the firms were indigenous construction firms and 13 (32.5%) were foreign construction firms. Table 2 showed the area of construction specialization of the indigenous and foreign construction firm. It showed that 17 (63%) of the indigenous firms specialize in building and civil engineering works with 10 (37%) in building works only while 11 (84.6%) of the foreign firms specialized in building and civil engineering works with 2 (15.4%) into building works only. Table 3 showed the sizes of the firms in terms of staff strength. The aggregation of the size of the Indigenous construction firms showed that 9 (33.3%) were small firms (less 25 staff), 9 (33.3%) were small to medium sized (25–100 staffs), 6 (22.2%) were medium sized (100 to 500) and 3 (11.1%) were large sized firms (500 & above) while there were no foreign firms in the small sized firm category, there were about 2 (15.4% )small to medium sized, 3 (23.1%) medium sized and majority with 8 (61.5%) were large sized firms. The primary responses were obtained from the management team of each construction firm based. The breakdown of the profession of the respondents in Table 4 showed that 5 (18.5%) were builders, 7 (25.9%) were architects, 11 (40.7%) were engineer and 4 (14.8%) were project managers in the indigenous construction firms. Likewise, the foreign construction firm’s respondent included 3 (23.1%) builders, 2 (15.4%) architects, 5 (38.5%) engineers and 3 (23.1%) were project managers. Table 5 showed the years of working experience in the construction industry of the respondents from each construction firm. In the indigenous construction firms, 7 (25.9%) had 1–5 years working experience, 10 (37%) had 6–10 years working experience, 8 (29.7%) of them had 10–15 years of working experience and 2 (7.4%) had 15–20 years of experience. Likewise, respondents in foreign construction firms, 3 (23.1%) had 6–10 years working experience, 5 (38.5%) had 10–15 years of experience and 5 (38.5%) had 15–20 years of experience. Table 6 showed the academic qualification of the respondents both in the indigenous and foreign construction firms. In Table 7, the mean scores and ranking index for the level of awareness of indigenous and foreign construction firms on sustainable development practices was presented. Table 7 when analyzed showed the comparison of the level of awareness of sustainable development practices and the overall mean score. Table 7 showed that foreign construction firms were mostly aware about conducting of frequent materials audits (5.00), protection of the environment and use of low or no VOC emitting paints and adhesives (4.92). A comparison with indigenous construction firms showed that they were more aware about protection of the environment (4.52), specifying and use of local materials which are sourced locally (4.33) and energy efficiency, using of alternative energy supplies (solar panel etc.) and installation of whole house ventilation systems (4.22). Further analysis of the dataset can show the dearth in awareness of crucial sustainable development practices in each firm type. Table 8 showed the mean score and ranking index on the consistency by the construction firms in the practice of sustainable development on their past and ongoing construction projects. It showed the extent to which each firm considers sustainable development practices as important. Indigenous construction firms consistently practice the protection of the environment (4.56), installation of water efficient fixtures (4.33) and waste management (4.30) on their construction project. Foreign construction firms on the other hand, were majorly concerned about conducting frequent materials audits, installation of water efficient fixtures and the protection of the environment. It is important that sustainable development practices are enshrined in the policy and commitment of construction firms. Therefore, the commitment would be determined by the different impact the firms hope to generate from their practices. Table 9 showed the mean scores and ranking index of the impact of implementing sustainable practices on the environment, firm, economy and client. Foreign construction firm perceived that sustainable development practices can enhance their corporate identity (4.54), increased profit and increased client base (4.46). For indigenous construction firms, the impact perceived from practicing sustainable development in their construction projects are client satisfaction (4.41) enhanced corporate identity and enhanced innovation (4.26). Further analysis of the dataset can show the underlying value indigenous and foreign construction firms place on sustainable construction practices (Table 4).

**Table 1:** Categories of firm.

**Type of firm**	**Frequency**	**Percentage %**
Indigenous	27	67.5
Foreign	13	32.5
Total	40	100.0

**Table 2:** Area of specialization of firms.

Firm Specialization	**Indigenous**		**Foreign**	
	**Frequency**	**Percent**	**Frequency**	**Percent**
Building works	10	37.0	2	15.4
Building and civil engineering works	17	63.0	11	84.6
Total	27	100.0	13	100

**Table 3:** Size of firms.

**Size of firm**	**Indigenous**		**Foreign**	
	**Frequency**	**Percentage%**	**Frequency**	**Percentage%**
Less than 25	9	33.3	0	0
25 to 100	9	33.3	2	15.4
100 to 500	6	22.2	3	23.1
500 & above	3	11.1	8	61.5
Total	27	100.0	13	100

**Table 4:** Professional background.

**Profession**	**Indigenous**		**Foreign**	
	**Frequency**	**Percentage**	**Frequency**	**Percentage**
Builder	5	18.5	3	23.1
Architect	7	25.9	2	15.4
Engineer	11	40.7	5	38.5
Project manager	4	14.8	3	23.1
Total	27	100.0	13	100

**Table 5:** Years of working experience.

Years	**Indigenous**		**Foreign**	
	**Frequency**	**Percentage**	**Frequency**	**Percentage**
1 - 5 years	7	25.9	0	0.0
6 - 10 years	10	37.0	3	23.1
10 - 15 years	8	29.7	5	38.5
15 -20 years	2	7.4	5	38.5
Total	27	100	13	100.0

**Table 6:** Academic qualification.

**Qualification**	**Indigenous**		**Foreign**	
	**Frequency**	**Percentage %**	**Frequency**	**Percentage %**
B.Sc/B.Tech	9	33.3	4	30.8
B.Eng	5	18.5	2	15.4
M.Sc	10	37.0	4	30.8
M.Phil/Ph.D.	3	11.2	3	23.1
Total	27	100.0	13	100

**Table 7:** Level of awareness on sustainable development practices.

**Sustainable practices**	**Indigenous**		**Foreign**		**Overall**
	**Mean Score**	**RI**	**Mean Score**	**RI**	
Protection of the environment	4.52	1st	4.92	2nd	4.65
Specify and use local materials sourced	4.33	2nd	4.77	8th	4.47
Energy efficiency	4.22	3rd	4.69	12th	4.37
Using Alternative energy supplies (solar panels e.tc)	4.22	3rd	4.92	2nd	4.45
Install whole house ventilation systems	4.22	3rd	4.85	5th	4.42
Appropriately dispose of waste water on site	4.19	6th	4.54	17th	4.30
Waste water management	4.15	7th	4.77	8th	4.35
Conduct frequent materials audits	4.15	7th	5.00	1st	4.42
Indoor Air quality control	4.11	9th	4.77	8th	4.33
Waste management	4.11	9th	4.85	5th	4.35
Install water efficient fixtures	4.07	11th	4.77	8th	4.30
Water conservation/ efficiency	3.93	12th	4.69	12th	4.18
Using recycled content materials	3.78	13th	4.62	15th	4.05
Use products or materials with recycled content	3.59	14th	4.38	18th	3.85
Restore ecosystems native plants	3.52	15th	4.85	5th	3.95
Use bio-based products or materials	3.37	16th	4.69	12th	3.80
Minimize use of PVC based products or materials	3.37	16th	4.62	15th	3.77
Use of low or no VOC emitting paints & adhesives	3.44	18th	4.92	2nd	3.95

*RI = Ranking Index

**Table 8:** Consistence in the practice of sustainable development practices.

**Sustainable practices**	**Indigenous**		**Foreign**		**Overall**
	**Mean Score**	**RI**	**Mean Score**	**RI**	
Protection of the environment	4.56	1st	4.69	3rd	4.60
Install water efficient fixtures	4.33	2nd	4.85	2nd	4.50
Waste management	4.30	3rd	4.38	10th	4.33
Install whole house ventilation systems	4.19	4th	4.69	3rd	4.35
Conduct frequent materials audits	4.15	5th	5.00	1st	4.42
Specify and use local materials sourced	4.15	5th	3.54	18th	3.95
Indoor Air quality control	4.11	7th	4.69	3rd	4.30
Appropriately dispose of waste water on site	4.07	8th	4.46	7th	4.20
Energy efficiency	3.89	9th	4.62	6th	4.13
Water conservation/ efficiency	3.81	10th	4.46	7th	4.02
Waste water management	3.70	11th	4.00	14th	3.80
Using Alternative energy supplies (solar panels e.tc)	3.67	12th	3.85	15th	3.72
Using recycled content materials	3.56	13th	4.46	7th	3.85
Minimize use of PVC based products or materials	3.44	14th	4.38	10th	3.75
Use of low or no VOC emitting paints & adhesives	3.41	15th	4.31	12th	3.70
Use products or materials with recycled content	3.30	16th	3.85	15th	3.48
Use bio-based products or materials	3.30	16th	3.62	17th	3.40
Restore ecosystems native plants	3.19	18th	4.08	13th	3.48

*RI = Ranking Index.

**Table 9:** Impacts of implementing of sustainable development practices.

**Impact**	**Indigenous**		**Foreign**		**Overall**
	**Mean score**	**RI**	**Mean score**	**RI**	
Enhancing corporate identity	4.26	2nd	4.54	1st	4.35
Increased profit	3.67	8th	4.46	2nd	3.93
Increased client base	4.19	4th	4.46	2nd	4.27
More cost incurred	3.65	9th	4.38	4th	3.90
Reduction of waste generation	4.08	6th	4.31	5th	4.15
Clients satisfaction	4.41	1st	4.31	5th	4.38
Increased time of project completion	4.15	5th	4.23	7th	4.18
Enhancing innovation	4.26	2nd	4.23	7th	4.25
Increased sales	4.04	7th	4.15	9th	4.07
Increased standard of living for employees	3.60	10th	4.10	10th	

*RI = Ranking Index.

## 2 Experimental design, materials and methods

The dataset was obtained from primary sources using the questionnaire instrument. The data article follows the works of previous studies in [6], [7], [8], [9], [10], [11], [12], [13], [14], [15], [16], [17], [18]. The questionnaire instrument was designed to have four (4) sections: the background information of the construction firms, the level of awareness, consistency in practice and the impact of practicing sustainable development on construction projects. The responses were based on a five-point Likert scale. The uniqueness of the dataset is the comparison of indigenous and foreign construction firms. The construction firms selected in this dataset were located in Lagos state. The state was selected due to its high volume of construction works due to its mega-city status of over 12 million people residing within the state. The sample size was selected using a purposive sampling method due to the characteristics of the construction firms. A total of twenty seven (27) indigenous construction firms and thirteen (13) foreign construction firms were selected for the dataset. Descriptive analysis such as frequencies, percentage and mean score were used to present the quantitative data in form of tables.
