# Supplementary material for: Dataset on sustainable construction practices of foreign and indigenous construction firms
Source: Data Brief. 2018 Aug 22;20:812–8. doi: 10.1016/j.dib.2018.08.044 (PMC6134159; doi:10.1016/j.dib.2018.08.044)
Supplement: Supplementary file 1 — Supplementary material [file mmc1.doc]

Article Title: ***Data Sets On*  Sustainable Construction Practices of Foreign and Indigenous Expatriate Construction Firms**

Declaration of interest

Response: None

Corresponding Author: Akinbo Faith Tomisin

Covenant University.
